# Progress in Gynæcology

**Published:** 1901-03-16

**Authors:** 


### Progress in Gynæcology.

**Dysmenorrhœa.**—Dr. Haultain[1] defines dysmenorrhœa as "pain associated with, and dependent on, menstruation of such severity as to materially interfere with the sufferer's performance of her usual duties." Practically the causes of dysmenorrhœa may be briefly considered as inflammatory and obstructive, alone or combined. The inflammatory variety embraced congestive and inflammatory conditions of the uterus and its appendages. The obstruction necessarily was confined to morbid states of the uterus alone. The pain in ovarian and tubal dysmenorrhœa was mainly pre-menstrual in its character, the depletion occurring as a result of the flow giving considerable relief. On the other hand, in uterine inflammation, such as endometritis, the pain though pre-menstrual, might be continued through the period by the contractions of the tender uterus. The treatment of dysmenorrhœa must be adapted to its many different causes: a point of prime importance is the question of the justification of the local treatment of dysmenorrhœa. With regard to married patients local examination, and if necessary treatment, should in all cases be carried out. With unmarried females, however, the lines of conduct are by no means so clear, but Haultain thinks that in these cases when, after a fair trial, drugs have failed to relieve the dysmenorrhœa, local examination under an anæsthetic is the correct mode of procedure, at the same time carrying out any treatment that may be required. The treatment of the menstrual variety of dysmenorrhœa by means of drugs is most discouraging. Haultain condemns the use of alcohol and morphine as likely to produce dipsomaniacs and morphomaniacs. In the inflammatory and congestive forms, particularly those due to ovarian causes, sedatives such as bromides, gelsemium, camphor, and hydrastis find most favour, combined with hot applications and counter-irritants. As regards operative treatment, he recommends dilatation of the cervix in those cases which are accompanied with congenital anteflexion and stenosis; and if endometritis is present as is so often the case, the uterus should be curetted. Incision of the cervix is not now so frequently adopted as formerly, but is often of much value in well marked cases of stenosis. He reserves oöphorectomy for those intractable cases in which every other resource has failed. Dr. Kerr[2] approves of dilatation of the cervix by the gradual method in preference to rapid dilatation. He has sometimes found electricity to be of service. Antipyrin and phenacetin were the only drugs he had found to be of any use. Dr. Macnaughton Jones[3] recommends senecin (2–6 grs.) either alone or with hydrastinine for amenorrhœa and dysmenorrhœa; in the congestive and spasmodic variety it should be combined with bromides. Dr. Church[4] maintains that more attention should be given to the preventive treatment of dysmenorrhœa. The present system of education among the cultured and educated classes made too severe a strain on the nervous system. To counteract this, greater attention should be paid to rest for a day or two during menstruation. When all due precautions had been taken to avert trouble, and there was yet dysmenorrhœa, moderate exercise on a cycle or horseback, between the periods, was sometimes useful.

**Malignant Disease of the Uterus.** — Professor Dmitri de Off[5] sums up his conclusions as regards the surgical treatment of cancer of the uterus as follows:—(1) Surgical treatment is now the sole means of successfully combating cancer of the uterus. (2) Total extirpation of the uterus must be performed in all cases of cancerous lesion of any part of the organ, as this procedure gives the best guarantee against recurrence. (3) Removal of the ovaries and appendages should be practised only when there are special indications. (4) The enucleation of retroperitoneal glands should be practised only by the abdominal route; it should be looked upon as a much more dangerous procedure. (5) Total extirpation of the uterus by the vaginal route is preferable to all other methods. (6) Morcellation is recommended in cases where the uterus is of considerable size, or where the case is complicated by the presence of fibroids. (7) In cases of pregnancy complicated with cancer of the uterus, extirpation is indicated: the vaginal route up to the seventh month, after that time, in view of the viability of the child, cœliotomy is the operation of choice. (8) The remote results of the surgical treatment of cancer of the body of the uterus are infinitely more favourable than those of cancer of the cervix. (9) Total extirpation of the uterus should be practised even in advanced cases, for by means of that operation one can most effectually relieve the patient, and postpone the fatal termination. H. J. Boldt[6] recommends vaginal hysterectomy in preference to the abdominal operation in malignant disease of the uterus. In comparing the merits of the two operations he says: Vaginal hysterectomy permits a smaller opening of the peritoneal cavity; it usually takes less time to perform, thus avoiding to a greater extent the element of shock; convalescence is more rapid, the abdominal wound with its occasional complications is avoided, and the direct mortality is smaller. He recommends that as much of the parametrium should be removed as possible, and in malignant disease of the vaginal portion, he resects a large portion of the upper part of the vagina, thus removing to a great extent the cancer-conveying channels. The advantage of the abdominal operation over the vaginal is that it permits the more extensive removal of the lymphatics, and of the iliac, retroperitoneal, and lumbar glands. These, however, he maintains, are rarely affected until late, when the disease has passed the boundaries of a radical operation. The only cases he considers in which the abdominal operation is indicated, are those in which the uterus is too large, or too adherent from inflammatory processes to be removed per vaginam without morcellation, and those in which the diagnosis of glandular enlargement is definitely made.

[1] Lancet, July 28, 1900. [2] Brit. Med. Jour., July 28, 1900. [3] Edin. Med. Jour., July, 1900, [4] Lancet, July 23, 1900. [5] Brit. Med. Jour., Sept. 1, 1900. [6] Amer. Gyn. and Obstet. Jour., Dec. 1900.

